# The Role of the Occupational Therapist in Disaster Areas: Systematic Review

**DOI:** 10.1155/2017/6474761

**Published:** 2017-01-31

**Authors:** M. Parente, M. Tofani, R. De Santis, G. Esposito, V. Santilli, G. Galeoto

**Affiliations:** ^1^Sapienza Università di Roma, Rome, Italy; ^2^Department of Anatomical, Histological, Forensic and Orthopaedic Sciences, Sapienza Università di Roma, Piazzale Aldo Moro 5, 00185 Rome, Italy

## Abstract

**Background:**

Disasters are increasingly more frequent events on our planet. During disaster the role of the occupational therapist will require a more specific operative framework within nongovernmental organizations and community health services.

**Design:**

Systematic review.

**Objective:**

The aim of this study is to evaluate the evidence that highlight occupational therapist's role in disaster area through a systematic review.

**Materials and Methods:**

Research on MEDLINE was performed. All articles from 2005 to 2015 concerning rehabilitation and occupational therapy in disaster areas were included.

**Results:**

Ten studies were selected to be included in this review. Four interesting points emerged: the importance of having rehabilitation intervention in postdisaster situations, the necessity to include a rehabilitation team in the early phase of disaster response, the need to provide a method to address the difficult evacuation, and finding the safest method of transport of people with preexisting disabilities and new injuries.

**Conclusions:**

The amount of evidence with respect to specific intervention of the occupational therapist's role in a disaster situation is limited. However some evidence suggests that it could be a good means for reducing the number of medical complications and deaths of persons with preexisting disabilities. The evidences found highlight the necessity to create a multidisciplinary team addressing needs in disasters situation, in which the occupational therapist could certainly contribute.

## 1. Introduction

Disasters are increasingly more frequent on our planet, even though their distribution is directly linked to the geophysical characteristics of the different regions.

According to the International Federation of Red Cross and Red Crescent Societies, disaster is a sudden, calamitous event that seriously disrupts the functioning of a community or society and causes human, material, and economic or environmental losses that exceed the community's or society's ability to cope based on its own resources [1]. Though often caused by nature, disasters can have human origins.

Usually, disasters are classified into two macrocategories: natural hazards and technological or man-made hazards. Natural hazards are naturally occurring physical phenomena caused either by rapid or slow onset events which can be geophysical (earthquakes, landslides, tsunamis, and volcanic activity), hydrological (avalanches and floods), climatological (extreme temperatures, drought, and wildfires), meteorological (cyclones and storms/wave surges), or biological (disease epidemics and insect/animal plagues). Technological or man-made hazards (complex emergencies/conflicts, famine, displaced populations, industrial accidents, and transport accidents) are events that are caused by humans and occur in or close to human settlements. These can include environmental degradation, pollution, and accidents. The combination of hazards, vulnerability, and inability to reduce the potential negative consequences of risk results in disaster.

**Figure 1:**
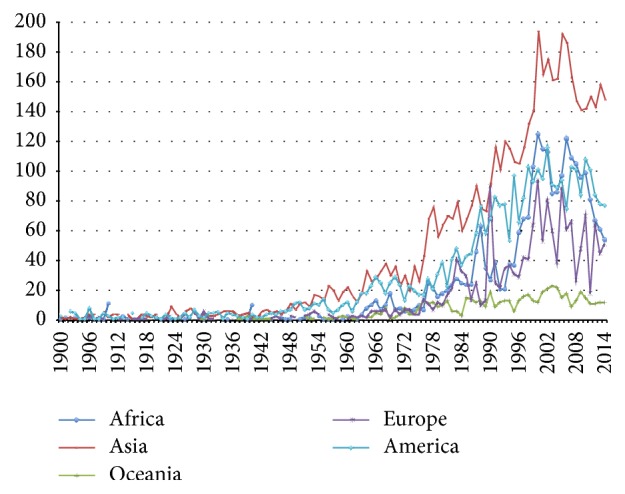
Number of natural disasters in the world from 1900 to 2014.

**Figure 2:**
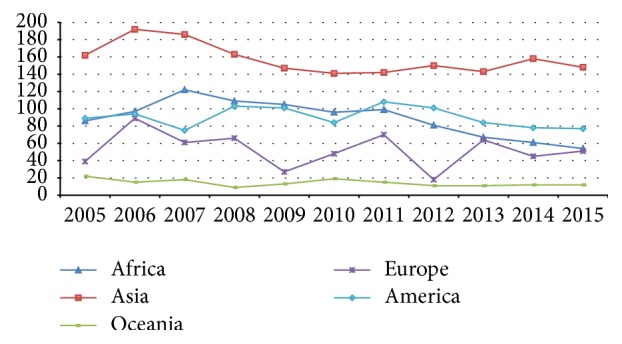
Number of natural disasters in the world from 2005 to 2015.

From 1900 to 2014 natural hazard exponentially increased (Figure 1); in particular during the last decade these phenomena have often been registered in different parts of the world, as indicated by Centre for Research on the Epidemiology of Disasters (CRED) (Figure 2) [2].

The effects of each disaster do translate not only into numbers of deaths, but also mostly into long-term disabilities that result from it. The most frequent disabilities are spinal cord injury, traumatic brain injury, fracture, limb amputation, peripheral nerve injury, and crush injury [3].

In the early stages after a disaster, humanitarian organizations and community health services have to schedule aids in emergency conditions. Furthermore, both volunteers and those associated with humanitarian organizations actually prove to be able to manage complex circumstances [4].

For this reason, a multidisciplinary emergency team should include rehabilitation professionals: as their competences and expertise could be useful to recognize and manage similar disabilities while trying to reduce the high risk of medical complications and, in turn, a possible aggravation unstable situation during the acute phase [4, 5]. Indeed the most urgent priority is to save lives and help people with disabilities who are at greater risk of dying or being left behind during the evacuation [6, 7]. Special attention should be given to these vulnerable categories of persons by rehabilitation professionals.

## 2. Objective

The aim of this study is to evaluate the evidences available in the literature that highlight the occupational therapist's role in a disaster situation through a systematic review.

## 3. Materials and Methods

Search terms included “rehabilitation”, “disaster”, “natural disaster”, and “occupational therapy”. Three independent searches were carried out using the following terminology: “disaster AND rehabilitation”, “natural disaster AND rehabilitation”, and “natural disaster AND occupational therapy”. All included studies had the following criteria: published in English on MEDLINE from January 2005 to September 2015. All articles about rehabilitation and occupational therapy interventions in disaster areas were included. Studies that did not have these characteristics were excluded. Titles and abstracts were read, articles not meeting selection criteria were discarded, and those remaining were read in full to check for suitability, in accordance with the Preferred Reporting Items for Systematic and Meta-Analyses (PRISMA).

Data extraction was completed by one reviewer confirmed by a fellow author. Relevant articles meeting the inclusion criteria were reviewed with all relevant information, such as type of design, participants' characteristics, and significant findings of outcome.

## 4. Results

**Figure 3:**
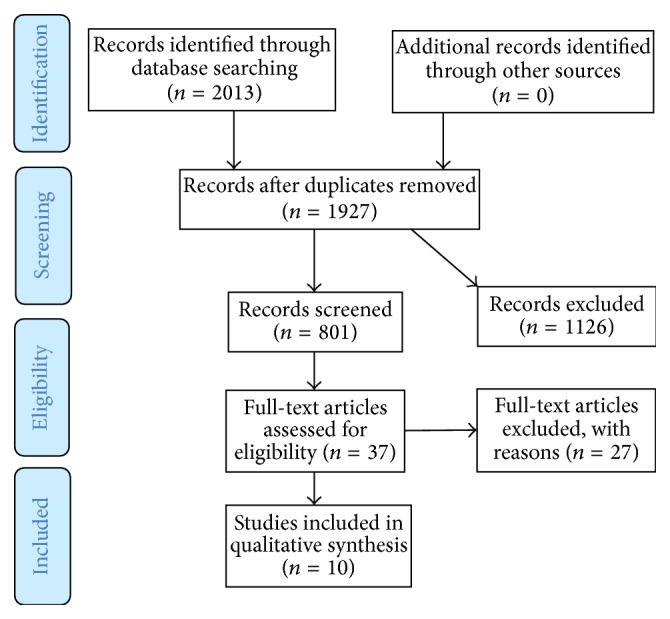
Flowchart.

The database search yielded 10 papers which met inclusion criteria (Figure 3).

All articles referred only to postearthquake disaster management. Search results have been summarized in Table 1. Four interesting points emerged: the importance of having rehabilitation intervention in postdisaster situations, the necessity to include a rehabilitation team in the early phase of disaster response, the need to provide a method to address the difficult evacuation, and finding the safest method of mobilization.

### 4.1. Rehabilitation in Postdisaster

Zhang et al. [12] studied the survivors to understand the motor functions and ADL capacity of patients with fractures sustained in the Wenchuan earthquake in 2008 and to provide a basis for rehabilitation and treatment. Fractures were the main issue in the seismic wounded; many of survivors had reductions in ROM, muscle force, and ADL capacities. Authors conclude that physicians involved in rehabilitation should pay greater attention to muscle force exercises, joint mobilization, and occupational therapy during the early phases after disaster.

Rauch et al. [14] described problems in functioning and associated rehabilitation needs in persons with spinal cord injury after the 2010 earthquake in Haiti by applying a newly developed tool based on the International Classification of Functioning, Disability and Health (ICF). This ICF-based needs' assessment provided useful information for rehabilitation planning in the context of natural disaster. Authors conclude that a multidisciplinary approach would be needed and in particular in low-resource countries it is crucial to enable local staff to perform assessments and provide education and training in rehabilitation management.

Reinhardt et al. [8] presented an evidence-based overview of the effectiveness of medical rehabilitation intervention in natural disaster survivors and outcomes that are affected. The findings suggest some evidence for the effectiveness of inpatient rehabilitation in reducing disability and improving participation and quality of life and in community-based rehabilitation program for participation. The findings also highlight the need to incorporate medical rehabilitation into response planning and disaster management for future natural catastrophes.

### 4.2. Rehabilitation during Early Phases of Response

Reinhardt et al. [8] have examined the role of health-related rehabilitation in disaster relief in terms of the epidemiology of injury and disability in natural disasters, the impact of natural disasters on health and rehabilitation systems, and the assessment of disability due to natural disasters. Authors also analyze the condition of persons with preexisting disabilities after a natural disaster: the loss of medications and assistive technologies can impair their quality of life. Furthermore the lack of trained support personnel can worsen the condition of these vulnerable persons. Authors conclude that significant systematic challenges to effective delivery of rehabilitation interventions during disasters include a lack of trained responders as well as a lack of medical recordkeeping, data collection, and established outcome measures.

Khan et al. [3] suggest that it is crucial to have support of a multidisciplinary team—including occupational therapist—in the early phase of a disaster. They also analyze the role of Physical and Rehabilitation Medicine and developed some recommendations including the necessity to develop scientific evidence for medical rehabilitation in the emergency disaster response, to develop a rehabilitation disaster relief expertise, and to strengthen an international rehabilitation emergency response capability where both International Society of Physical and Rehabilitation Medicine and other rehabilitative associations could cooperate.

Landry et al. [11] analyzed the situation that occurred after earthquake in Haiti in 2010 as an important inflection point for international humanitarian efforts that target rehabilitation. Authors analyzed problems related to organizing early intervention in a low-resources country based on treatment of disabling consequences and based on relieving the stress of local health care systems. In this article authors highlighted the need to incorporate rehabilitation into response planning for future humanitarian catastrophes.

### 4.3. The Issues of Persons with Preexisting Disabilities

Liu et al. [10] focused their intervention in particular to prevent immobilization syndrome and progressive functional deterioration among frail elderly survivors and persons with preexisting disabilities who were forced to stay in shelters that were not designed to encourage physical activity. Authors evaluate activities of 10 rehabilitation-related organizations, which include the occupational therapist. This first collaborative disaster relief endeavour by rehabilitation-related organizations and professionals has contributed to a strong foundation for future interdisciplinary and interorganizational collaborative activities.

Hunt et al. [9] contribute to better understanding of the perceptions of responders and decision-makers regarding disability and efforts to address the needs of persons with disabilities following the 2010 earthquake in Haiti. In fact, following disasters, persons with disabilities are especially vulnerable to harm, yet they have commonly been excluded from disaster planning, and their needs have been poorly addressed during disaster relief.

### 4.4. The Need of a Correct Mobilization and Transport for the Newly Acquired and Old Disabilities

Gosney et al. [5] in this narrative review collected data about spinal cord injury (SCI), one of the most frequently occurring disabilities after a hazard. Authors discuss the correct managing of SCI and underline the importance of correct mobilization of new acquired injury. This article strengthens the idea of the necessity of a multidisciplinary approach.

Rathore et al. [13] shed light on some crucial aspects that emerged during the first aid phases in the 2005 Pakistan earthquake by summarizing the services provided. Above all, the issue of evacuation and transport of the newly acquired SCI was addressed. Authors highlighted the lack of first response teams working in the emergency after disaster to correctly evacuate, safely mobilize, and transport people with SCI.

## 5. Discussion

Articles focusing on occupational therapy were not found. However, different experiences of occupational therapists were cited emphasizing their appropriateness due to professional training. After our analysis, it seems fitting that occupational therapist may be eligible into being a part of the response team in a natural disaster.

According to the American Occupational Therapy Association's (AOTA) position paper regarding the role of occupational therapy in disaster preparedness, response, and recovery, five stages of disaster relief were categorized:

- preimpact;
- impact;
- immediate postimpact;
- recovery;
- reconstruction [15].

Considering preimpact and impact stages, occupational therapist may find his competencies more useful to organize in collaboration with multidisciplinary team such as an evacuation plan for people affected by preexisting disabilities and planning access to first aid facilities, which are not always accessible for people with mobility impairment.

Occupational therapists are also widely involved in immediate postimpact, recovery, and reconstruction stages. They can work both in subacute rehabilitation treatment facilities and promoting the reintegration of the individual into family and society. The immediate role of the occupational therapist in the postimpact phase is not well defined in the literature; evidence suggests the necessity to create a multidisciplinary network to better organize technical operations.

In Rathore et al.'s study “Rehabilitation during Early Phase of Response” focused on what physiatrists can do during early phases of intervention. Authors confirm the necessity to create a multidisciplinary international network to manage emergency operations.

In the “Rehabilitation in Post Disaster” section the authors highlight the importance of evaluation of any assistive technologies needed, such as wheelchairs. Furthermore mobilizing preexisting or newly acquired disabilities require professional skills and a trained therapist or volunteer in order to reduce the risk of wrong and hazardous movements.

In the “The Issues of Persons with Pre-Existing Disabilities” section the authors describe the importance of a communitarian approach to improve rehabilitation and prevent progressive functional deterioration of people with disabilities. Authors stressed the importance to define an operative framework within community health services.

Therefore, we believe that the occupational therapist can play a significant role in different situations.

## 6. Study Limitations

One of the most important limitations that our study encountered was the low number of scientific evidence and the lack of material on this topic in the literature. In fact there were no articles in which occupational therapy was the centre of the rehabilitation interventions. Furthermore, lots of studies were conducted in very poor areas, such as Haiti, Pakistan, and the countryside of China, where health and rehabilitation networks are not well developed.

## 7. Conclusions

In conclusion, this study had the objective of verifying evidence about occupational therapist intervention [16, 17] in a disaster area. We can state that occupational therapist could reduce complications' rate and worsening of medical case.

The ten articles included talked about the necessity of a rehabilitation team in providing measures to reduce damage in early postimpact phases. The occupational therapist could take an active role in emergency management, as follows:

- They are important members of any rehabilitation team and can provide vital service in the early phase of response.
- They have expertise in caring for preexisting disabilities.
- They have expertise in problem solving to correct mobilization and evacuation, while taking into consideration preexisting and newly acquired conditions.

Therefore, investing and introducing occupational therapists in first response teams could not only translate into a reduction of medical complications and a better quality of life for people with disabilities, but could also translate into potential economic benefits as cutting the cost for care additional services and assistance from National Health Systems.

Another aspect that we might consider is the possibility of using an occupation therapist as an instructor to provide necessary education to multidisciplinary team and as a volunteer who helps in emergency management.

## Figures and Tables

**Table 1:** Data extraction.

References	Objectives	Study type	Participants' characteristics	Results	Conclusions
Reinhardt et al. [8], 2011, Global Health Action	To examine the role of health-related rehabilitation in natural disaster relief along three lines of inquiry: epidemiology of injury and disability, impact on health and rehabilitation systems, and the assessment and measurement of disability.	Qualitative literature review	*N* = 246 deaths and affected by year and region *N* = 568 deaths in earthquake *N* = 41 deaths and affected in Asia	Major impairments requiring health-related rehabilitation include amputations, traumatic brain injuries, spinal cord injuries (SCI), and long bone fractures. Studies show that persons with preexisting disabilities are more likely to die in a natural disaster.	Additional development of health-related rehabilitation following natural disaster is urgently required.

Hunt et al. [9], 2015, Global Health Action	To better understand the perceptions of responders and decision-makers regarding disability and efforts to address the needs of PWD following the 2010 earthquake.	Qualitative study	*N* = 24 persons involved in the national and international first aid associations; 11 women; 13 men	Participants identified PWD as being among the most vulnerable individuals following the earthquake. Though some forms of disability received considerable attention in aid efforts, the needs of other PWD did not. Several factors were identified as challenges for efforts to address the needs of PWD including lack of coordination and information sharing, the involvement of multiple aid sectors, perceptions that this should be the responsibility of specialized organizations, and the need to prioritize limited resources.	There have been several efforts to promote best practices and develop guidelines to better address the needs of PWD in disasters; significant obstacles remain to the implementation of disaster preparedness, relief, and reconstruction that is inclusive of PWD and responsive to their needs.

Khan et al. [3], 2015, Archives of Physical Medicine and Rehabilitation	To present an evidence-based overview of the effectiveness of medical rehabilitation intervention in natural disaster survivors and outcomes that are affected.	Systematic review	*N* = 10 studies (2 randomized controlled trials, 8 observational studies) *N* = 2013 participants; age 9–76, mostly women	There are some evidence for the rehabilitation short and long-term improvement in terms of functional activity, psychological symptom, and participation. More attention must be paid to the rescue of preexisting disabilities.	The findings highlight the need to incorporate medical rehabilitation into response planning and disaster management for future natural catastrophes. Access to rehabilitation and investment in sustainable infrastructure and education are crucial. More methodologically robust studies are needed to build evidence for rehabilitation programs, cost-effectiveness, and outcome measurement in such settings.

Gosney et al. [5], 2013, Spinal Cord	To summarize epidemiological and scientific research on spinal cord injury (SCI) populations from three severe earthquakes (EQs) in rehabilitation resource-scarce settings; summarize SCI rehabilitation services by local and foreign providers; and provide implications including research gaps for a supporting global scientific research agenda.	Narrative literature review	*N* = 11 studies (4, 2005 Pakistan earthquake; 4, 2008 China earthquake; and 3, 2010 Haiti earthquake)	The range of long-term disabilities is more than death's range. Sometimes the rescue operation of SCI patients is not accurate; therefore the clinical picture is made worse.	A global disaster research agenda for SCI in EQs in rehabilitation resource-scarce settings is needed to strengthen the evidence base for improvement of clinical management and outcomes for SCI EQ survivors.

Liu et al. [10], 2012, Journal of Rehabilitation Medicine	To provide descriptive epidemiology and assess the activities of 10 rehabilitation- related organizations.	Descriptive	10 rehabilitation-related organizations	10-RRO provided relief activities at 3 shelters. Support activities included prevention of immobilization, daily life support, environmental improvement, and transition to temporary housing. The questionnaire survey revealed poor preparedness, satisfactory initial response and support activities, and problems of data collection and advocacy.	The disaster was characterized by minimal trauma and a great need for preventing immobilization. There is an urgent need to develop such a manual to improve preparedness and enhance capability of first aid team to cope with disasters.

Landry et al. [11], 2010, Disability and Rehabilitation	To underline the role of rehabilitation during and after Haiti earthquake	Report	Undefined	There is a remarkable increment of permanent disabilities caused also by attempt of rescues. For this reason there is a tremendous need of rehabilitation services.	The events have raised awareness of the importance of rehabilitation services and highlighted the need to incorporate rehabilitation into response planning for future humanitarian catastrophes.

Zhang et al. [12], 2011, Chinese Medical Journal	To value disability impact on motor function and ADL	Retrospective cohort study	*N* = 395 patients; 218 men, 55.2% fractures; 117 women, 44.8% fractures;	Most survivors 82% had decreased ROM and 23.5%. Muscle force 72.2% had also restricted ADL capacities. With time the ADL capacities of female patients increased compared to the male patients.	Fractures were the main issue among the injured. Many patients had decreased ROM, ADL capacities, and muscle force; this highlights that physician, involved in rehabilitation, should pay great attention to muscle force exercises, joint mobilization, and occupational therapy during the early phase after disaster.

Rathore et al. [13], 2008, Archives of Physical Medicine and Rehabilitation	To sum up the interventions, the gaps, and the needs emerged the day after the 2005 earthquake in Pakistan.	Report	The ratio of males to females injured 1 : 1.3 Mean age 28, 16.5% less than 18 y; 89% paraplegia cases	Spinal trauma is a surgical emergency that requires specialized care in the initial immobilization and transport of a patient. Unfortunately, after the earthquake, there was usually such little care taken in transporting patients with a suspected or diagnosed SCI. Many physicians involved in the care of SCI patients were unaware of the ASIA system and its worksheet documentation. This resulted in errors in the diagnosis of complete and incomplete SCI.	There is a need to increase disaster preparedness and have a tangible disaster management plan in place and periodic disaster drills. Trauma management in disasters and the correct SCI evacuation, immobilization, and transport protocols should be taught during the training of emergency relief workers, ambulance officers, army medical staff, resident surgeons, and emergency physicians. Rescue units trained in methods to avoid and minimize spinal injuries should be established.

Rathore et al. [4], 2012, Archives of Physical Medicine and Rehabilitation	To stimulate development of research and practice in the emerging discipline of disaster rehabilitation within organizations that provide medical rehabilitation services during the postdisaster emergency response.	Report	Undefined	Medical rehabilitation is an urgent, essential emergency medical service in disasters and not restricted to the intermediate and long-term care settings. Emergency rehabilitation services should only be provided by trained, credentialed professionals to ensure practice accountability and proper standards of care. Nonqualified personnel, although well intentioned, should provide care only in the event of extreme emergency and under strict supervision.	Evidence on the effectiveness of disaster rehabilitation interventions is presented; indeed these services can reduce morbidity and improve functional results and survival.
